# Decoding the periodontitis–coronary artery disease axis through biomarker profiling: a systematic review and meta-analysis

**DOI:** 10.1186/s12903-026-08407-2

**Published:** 2026-04-27

**Authors:** Rahayu Zulkapli, Nusaibah Sallehuddin, Nashuha Omar, Norazreen Zakaria, Adriana Hamiera Zainol Anuar, Iman Nabilah Abd Rahim, Mohd Yusmiaidil Putera Mohd Yusof

**Affiliations:** 1 Cardiovascular Advancement and Research Excellence Institute (CARE Institute), Universiti Teknologi MARA, Jalan Hospital, Level 4, Academic Building, Sungai Buloh, Selangor 47000 Malaysia; 2 Faculty of Dentistry UiTM Campus Sungai Buloh, Universiti Teknologi MARA (UiTM), Jalan Hospital, Sungai Buloh Campus, Sungai Buloh, Selangor 47000 Malaysia; 3 Faculty of Applied Science, UiTM Campus Shah Alam, Universiti Teknologi MARA (UiTM), Shah Alam Campus, Jalan Ilmu 1/1, Shah Alam, Selangor 40450 Malaysia

**Keywords:** Periodontitis, Cardiovascular diseases, Coronary artery disease, Biomarkers

## Abstract

**Objectives:**

This systematic review and meta-analysis aimed to explore the biomarker profiles in adults diagnosed with periodontitis (PD) and coronary artery disease (CAD). Identifying shared biomarkers is crucial for clarifying the biological link between these two common inflammatory diseases, improving early risk detection, and supporting more integrated approaches to managing oral and cardiovascular health.

**Method:**

A comprehensive literature search was conducted in Scopus, PubMed, and Web of Science, covering studies published up to December 2024, in accordance with Cochrane handbook guidelines. The study population consisted of adults diagnosed with PD and either confirmed CAD or cardiovascular risk indicators. The eligibility criteria required original full articles in English that assessed quantitative biomarker levels in individuals with both conditions compared to controls. The study design included observational (*n* = 14) and randomized controlled trials (*n* = 6). A random-effects meta-analysis was performed, and heterogeneity was assessed using the I² statistic.

**Results:**

A total of 3,245 records were identified from three databases: PubMed (*n* = 737), Scopus (*n* = 1241), and Web of Science (*n* = 1267). The most frequently reported biomarkers among individuals with both PD and CAD were C-reactive protein (CRP/hs-CRP) and interleukin-6 (IL-6), followed by interleukin-1β, interleukin-8, and tumour necrosis factor-alpha. Five studies were included in the primary pooled meta-analysis for CRP analyses, that showed a significant pooled effect (SMD = 0.501; *p* = 0.001), while three studies stated that IL-6 showed no significant association (SMD = 0.166; *p* = 0.240). Overall, studies demonstrated elevated systemic inflammatory biomarkers in individuals with both conditions.

**Conclusions:**

The findings suggest a potential association between chronic PD and elevated systemic inflammatory biomarkers in individuals with CAD. In particular, CRP was consistently reported across several studies, although the strength and significance of biomarker associations varied. These results indicate that periodontal inflammation may contribute to systemic inflammatory responses relevant to cardiovascular pathology; however, current evidence remains insufficient to establish causality. Further well-designed longitudinal and interventional studies are required to clarify the clinical significance of this relationship.

**Supplementary Information:**

The online version contains supplementary material available at 10.1186/s12903-026-08407-2.

## Introduction

Periodontitis (PD) is the inflammation of the periodontium, which is the supporting structure of the teeth, including the periodontal ligament and alveolar bone [1]. The disease is characterised by gingivitis at an early stage, deterioration of the periodontal ligament and dental cementum, loss of clinical attachment and alveolar bone, deep probing depths, and bleeding upon probing [1]. The most common characteristic of PD is the resorption of alveolar bone [2].

Periodontitis may present in different clinical forms depending on disease progression and underlying pathology. According to the periodontal disease classification proposed by Armitage, periodontitis is primarily categorised into chronic periodontitis and aggressive periodontitis, with chronic periodontitis representing the most common form and typically characterised by slow to moderate progression of periodontal destruction [3]. In contrast, acute periodontal conditions generally refer to specific entities such as necrotizing periodontal diseases or periodontal abscesses, which present with a rapid onset of symptoms and pronounced inflammation and are clinically distinct from chronic plaque-associated periodontal destruction [3].

PD patients may exhibit reduced mastication [1]. The aetiology of PD involves the triad of specific bacterial pathogens, destructive host immune responses, and environmental factors [1]. Bacteria, especially anaerobes, produce toxins and enzymes, which stimulate the innate and adaptive immune systems [2]. The activation of toll-like receptors stimulates the release of proinflammatory cytokines, such as interleukin (IL)-1, IL-6, and tumour necrosis factor-alpha (TNF-α) [4]. Approximately 11% of the world population, amounting to 743 million individuals, have severe PD [1]. Furthermore, PD is diagnosed more in the elderly compared to younger individuals [5]. Risk factors for PD include poor oral hygiene, smoking, drugs, alcohol abuse, mental instability, metabolic diseases, systemic inflammation, and socioeconomic status [5]. Studies found that PD stems from the inflammatory response by the host at both the local and systemic levels [2]. In addition, another study found that childhood socioeconomic status could have irreversible impacts on adulthood oral health [5]. Findings from case-control and cross-sectional studies imply that PD may lead to tooth loss, systemic inflammation, premature babies, and cardiovascular diseases (CVD) [6]. Further research suggested another pathophysiology includes microbial load and homeostatic disturbances [7]. Ultimately, these studies suggest a potential connection between oral and overall health.

CVDs are a group of disorders of the heart and blood vessels in which patients remain asymptomatic for a long time [8]. Examples of CVD are coronary artery disease (CAD), hypertension, ischemic heart disease, heart failure, cardiomyopathy, arterial fibrillation, and stroke [9]. CAD is characterised by lipid buildup, or atherosclerosis, in the coronary arteries, leading to inadequate blood and oxygen supply to the myocardium [10]. CAD patients may experience a variety of symptoms, from chest pain to asymptomatic. Risk factors for CAD include sedentary lifestyle, smoking, obesity, and high blood pressure [11]. CAD is the most common cause of death, accounting for one-third of mortality in individuals older than 35 years of age, and the risk of death increases significantly with age [12]. Nevertheless, compared to the earlier generation, the mortality rate has decreased over the last 40 years [10]. CAD mortality rates differ between developed and developing countries, in which the latter reported approximately 7 million deaths and 129 million DALYs annually [12]. Furthermore, race plays a role in which the mortality rates are found to be higher in Black people compared to White people [12]. CAD is also the most common cause of loss of Disability Adjusted Life Years (DALYs) [10].

The potential association between periodontal diseases, such as PD and gingivitis, and CVD, such as CAD and aortic aneurysm, has been identified in various studies [1]. For example, Sanikop et al. (2022) found that cross-sectional studies of patients with CAD had significantly higher PD than controls [7]. Localised inflammation of the periodontium may lead to systemic inflammation, which can contribute to atherosclerosis and other cardiovascular issues [6]. Bacteria from the periodontium, such as *Streptococcus sanguis* and *Porphyromonas gingivalis*, enter the bloodstream and exacerbate immune responses, elevating systemic inflammatory markers such as C-reactive protein (CRP) and IL-6, which are also linked to atherosclerosis [6]. Inflammatory cytokines promote endothelial dysfunction, facilitating the recruitment of circulating monocytes into the arterial wall, where they differentiate into macrophages and internalise oxidised low-density lipoprotein (ox-LDL), forming foam cells [13]. Foam cells produce various inflammatory proteins, including matrix metalloproteinases (MMPs), IL-6, and CRP, further exacerbating systemic inflammatory responses [11]. Furthermore, the invasion triggers platelet aggregation, increased blood viscosity, thrombus formation, and the activation of foam cells that ingest low-density lipoprotein (LDL), resulting in the thickening of the arterial wall and narrowing of the lumen [7]. The immune response to bacterial infection is influenced by genetic and environmental factors [5]. Recent studies have established the relationship between increased CRP, fibrinogen, lipid profile, white blood cells, IL-1β, IL-6, and TNF-α with the severity of CAD [14]. Furthermore, unhealthy lifestyles such as smoking increase the association of PD and CAD by weakening the immune response to infections, promoting chronic inflammation, and impairing blood circulation [6]. In addition, scientific evidence revealed the presence of genetic factors in both PD and CAD [15].

The purpose of this study was to review the association between PD and biomarker profiles in adults diagnosed with CAD. A clearer understanding of these shared biomarkers may support earlier risk detection, more targeted preventive strategies, and improved overall patient outcomes.

## Methods

**Fig. 1:**
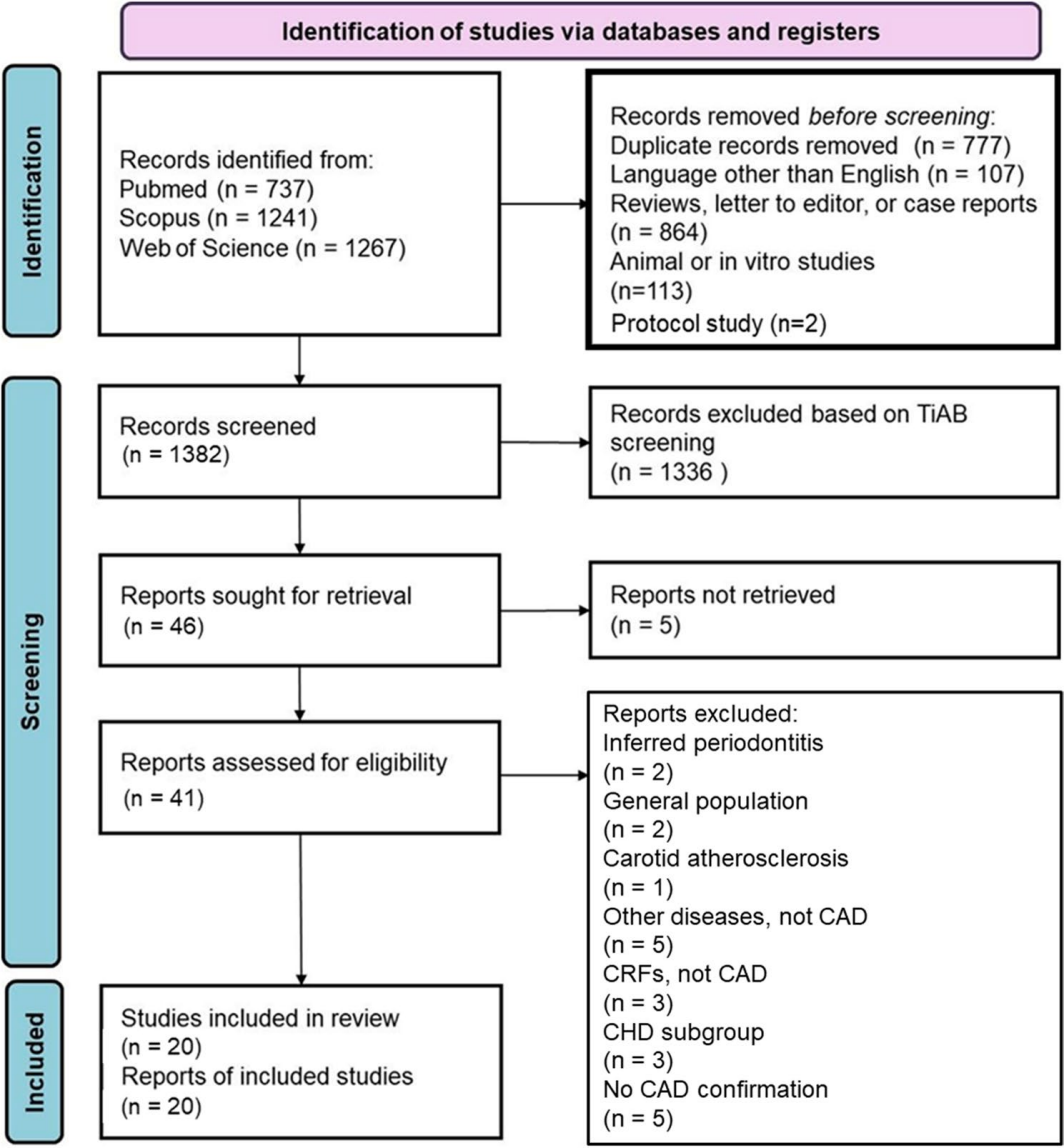
The PRISMA 2020 flow chart

This systematic review has been registered with the National Institute for Health Research PROSPERO, International Prospective Register of Systematic Reviews (PROSPERO No.: CRD420251036959). This review was conducted based on the Cochrane Handbook of Systematic Reviews of Interventions [16] and reported according to the Preferred Reporting Items for Systematic Reviews and Meta-Analyses (PRISMA) [17] (Fig. 1).

We developed a protocol to address the following PI(E)CO question:

What are the differences in the biomarker levels in adults diagnosed with PD and CAD or cardiovascular disease–related risk indicators compared to controls?

Each question had the following statements:

- P (Population): Adults (30–65 years) attending cardiology / dental clinics
- I (Intervention/Exposure): Adults with PD and either confirmed CAD or cardiovascular disease–related risk indicators
- C (Comparison): Individuals without PD and/or without CAD or cardiovascular disease–related risk indicators, or individuals with different periodontal status
- O (Outcome): Differences in quantitative biomarker levels (e.g., CRP, IL-6, TNF-α, and other inflammatory biomarkers) measured in serum, plasma, saliva, or other biological samples

### Search strategy

A systematic literature review was conducted to identify relevant studies investigating biomarkers that correlate PD with CAD in clinical studies. The research question for this study is: What biomarkers correlate PD with CAD in clinical studies among adults?

On 15 December 2024, we searched Scopus, PubMed, and Web of Science for studies published up to December 2024 that examined biomarkers linking chronic PD with cardiovascular diseases. The complete database-specific search strategies are provided in Supplementary Table S1. The key search terms used were (periodontitis OR “periodontal disease*” OR “gum disease*”) AND (“coronary artery disease” OR CAD OR “ischemic heart disease” OR IHD OR “coronary heart disease” OR CHD OR atherosclerosis) AND (biomarker* OR “inflammatory marker*” OR " biomarker " OR miRNA* OR “gene expression” OR proteomic*).

### Inclusion criteria

Articles were included if the following criteria were fulfilled: (1) the study assessed the association between biomarkers and either chronic or acute PD in clinical studies involving patients with CAD or cardiovascular disease-related risk indicators; (2) the study involved adult patients diagnosed with PD (either chronic or acute) and CAD or cardiovascular disease–related risk indicators and included a comparison group (e.g., individuals without PD, without CAD, or with different periodontal status); (3) the study focused on identifying biomarkers such as DNA-based markers or inflammatory markers (e.g., CRP, IL-6, TNF-α) linking PD to CVD or related inflammatory risk; (4) it was an original full article; and (5) studies published in English.

### Exclusion criteria

Articles were excluded from the review if: (1) it was a review article, editorial, commentaries, case reports, or letters to the editor; (2) the study involved animal or in vitro experiments; (3) the study focused on other oral diseases unrelated to PD; (4) the study did not report original data or was presented only as an abstract; or (5) the study did not examine biomarkers related to both PD and CAD or cardiovascular disease–related risk indicators.

### Study identification and selection

All citations identified from the search were downloaded into EndNote X7 software. The citations were organised, and duplicates were identified and removed. Two independent reviewers conducted the study selection process. In case of discrepancies in the selection process, a third reviewer was consulted to confirm the inclusion or exclusion of articles.

### Data extraction

Articles published up to December 2024 were selected. Initially, based on the titles and abstracts, articles that did not meet the inclusion criteria were excluded. Subsequently, a full-text review was conducted, and articles that did not meet the inclusion criteria were excluded. In the case of duplicate publications, only the most recent version was included. The following data were extracted from the selected studies: (1) the aim of the study, (2) a brief description of the subjects, (3) methods of data collection and analysis, (4) type of biomarkers studied, (5) results related to the association between biomarkers, chronic PD, and CAD, and (6) the conclusions of the study.

### Review method

All potential articles were identified and screened by two independent reviewers. Study selection and data extraction were conducted after a detailed discussion, and any disagreements or uncertainties regarding study inclusion were resolved through consensus.

### Data analysis

Descriptive analyses of the study characteristics and statistical methods used were performed. This review is descriptive, and all results are presented as frequency counts to determine the number of studies that found a positive or negative correlation between biomarkers, chronic PD, and cardiovascular diseases. A summary table presents the identified biomarkers and their association with chronic PD and CAD (Table 1).

**Table 1:** Summary of selected studies of biomarker profiles in adults with CAD and chronic PD

Type of Study	Study ID	Sample Size	CAD Diagnosis	PD Diagnosis	Assay and Sample type	Statin/ Aspirin Use	Common Comorbidities	Type of Biomarkers Studied
Observational	Sanikop (2022) [7]	100	≥ 50% stenosis in ≥ 1 coronary artery on angiography, history of MI or stable angina. [Phenotype: Stable]	> 10 sites with PD ≥ 5 mm and CAL ≥ 4 mm; diagnosed using GI and RPI indices; ≥ 14 natural teeth. [Chronic PD]	Blood [Biochemical Assay]	NM	None (diabetes, hypertension, and smoking excluded)	Fibrinogen↑ CRP↑
	Bagavad Gita (2019) [18]	264	Acute Coronary Syndrome confirmed by ECG changes, cardiac enzymes (cTnI), and clinical findings. [Phenotype: Acute]	Diagnosed per Eke et al. (2012) criteria (CAL ≥ 4 mm and PPD ≥ 5 mm in ≥ 30% of sites). [Chronic PD]	Subgingival plaque [qPCR]	NM	Diabetes, hypertension, and smoking were excluded or adjusted as confounders	miR-146a↑ IL-6 ↑ TNF-α↑ IL-1β↑
	Sebring (2024) [19]	1,610	First myocardial infarction confirmed clinically and by SWEDEHEART registry. [Phenotype: Acute]	Primary AP = non–root-filled teeth; secondary AP = root-filled teeth with periapical lesion. [Chronic PD]	Blood [ELISA]	97% patients were prescribed statins and aspirin	Smoking Diabetes mellitus Hypertension	Primary AP: IL-8↑ Root-filled teeth: hs-CRP↑ Fibrinogen↑ Leukocytes↑ IL-1β↓ IL-2↓ IL-6↓ IL-12p70↓
	Górska et al. (2019) [20]	417	Acute Myocardial Infarction (MI) confirmed per ESC guidelines (24.9% STEMI, 75.1% NSTEMI). [Phenotype: Acute]	Chronic PD (CP) was evaluated clinically using PI, BoP, PPD, CAL, and Community Periodontal Index. [Chronic PD]	Blood [Biochemical Assay]	Yes	Smoking Hypertension Diabetes Peripheral arterial disease	CRP↑ Fibrinogen ↔ NT-proBNP ↔
	Sitompul et al. (2023) [21]	80	≥ 70% coronary stenosis confirmed by angiography. [Phenotype: Stable]	Based on CAL and PPD; ≥ 20 natural teeth; classified as mild or moderate–severe CP. [Chronic PD]	Blood [ELISA]	NM	Hypertension Diabetes Mellitus Obesity Smoking Dyslipidemia	IL-6 → CRP ↑ (significant correlation)
	Ilango (2023) [22]	240	Confirmed by coronary angiography (≥ 50% stenosis in ≥ 1 artery) or history of MI/stable angina; CABG samples used for PTX3 measurement. [Phenotype: Stable]	≥ 10 teeth; diagnosed per 2017 World Workshop criteria (Stage III/IV CP); assessed with PI, BI, PPD, CAL indices. [Chronic PD]	Subgingival plaque and atheromatous plaque [qPCR]	NM	Diabetes, hypertension, and other systemic conditions excluded	Pentraxin-3↑ IL-6 ↔ CRP ↔
	Teeuw et al. (2015) [23]	171	None – explored genetic overlap with atherosclerotic CVD risk locus (ANRIL, 9p21). [Phenotype: Stable]	Moderate–severe PD based on CDC–AAP case definition (Eke et al., 2012). ≥2 interproximal sites with CAL ≥ 4 mm on different teeth + radiographic bone loss. [Chronic PD]	Plasma [High sensitivity nephelometric method]	NM	Systemic diseases excluded (no diabetes, CVD, infections, or medications); adjusted for age, gender, BMI, and smoking	CRP↑
	Joseph et al. (2011) [24]	176	None – CVD excluded. study examined homocysteine as a cardiovascular risk biomarker in periodontitis). [Phenotype: Stable]	Chronic PD classified into moderate and severe using CDC–AAP (Page & Eke, 2007) definition — based on CAL and PD (Moderate: ≥2 sites with CAL ≥ 4 mm or PD ≥ 5 mm; Severe: ≥2 sites with CAL ≥ 6 mm and ≥ 1 site with PD ≥ 5 mm). [Chronic PD]	Plasma [Immuno- assay]	NM	Excluded systemic disease (CVD, renal disease, diabetes, rheumatoid arthritis, pregnancy/lactation); all nonsmokers with matched BMI and lipid profile	Plasma Homocysteine↑
	Flores (2014) [25]	93	Stable CAD (≥ 6 months post-diagnosis) confirmed by cardiology evaluation; outpatients with previous MI, stable angina, or ischemia on non-invasive tests. [Phenotype: Stable]	Periodontal parameters: BoP, CAL, PD measured at 6 sites/tooth; severe PD defined as ≥ 30% of sites with CAL ≥ 6 mm. [Chronic PD]	Blood [Biochemical Assay]	Majority were prescribed statins	Hypertension Diabetes (controlled), Smoking	CRP↑
	Fedele (2011) [26]	17,223	Self-reported history of myocardial infarction, angina, stroke, congestive heart failure, and clinically measured high BP (≥ 130/85 mmHg). [Phenotype: Stable]	Oral mucosal diseases - clinically diagnosed (infectious, inflammatory, traumatic, neoplastic lesions). PD was recorded as a covariate but not the main focus of analysis. [Chronic PD]	NM [Biochemical Assay]	NM	Hypertension Diabetes Dyslipidemia Smoking Obesity	hs-CRP ↑ Fibrinogen ↑
	Zanella et al. (2012) [27]	206	Coronary angiography (ACC National Cardiovascular Data Registry criteria; gold standard for CAD diagnosis). [Phenotype: Stable]	Chronic PD, diagnosed clinically by a periodontist through visual/tactile examination of plaque, calculus, bleeding, exudate, and inflammation; tooth loss is used as an indicator of disease severity. [Chronic PD]	Blood/ Serum [Biochemical Assay]	NM	Hypertension Diabetes mellitus Dyslipidemia	Inflammatory biomarkers (CRP & leukocytes) did not differ between CAD and non-CAD groups
	Miyoshi et al. (2018) [28]	1815	Serum hs-CRP (systemic inflammation indicator, ≥ 1 mg/L = high CVD risk). [Phenotype: Stable]	Periodontal examination (probing depth, bleeding on probing, mean PD). [Chronic PD]	Saliva/ Serum [Immuno- assay]	NM	Hypertension Diabetes mellitus Dyslipidemia Overweight (BMI ≥ 25)	Salivary LDH↑ hs-CRP↑
	Rizzo et al. (2012) [29]	44	No clinical CAD diagnosis: the study explored biochemical risk indicators (Hsp60, dyslipidemia) rather than confirmed CAD. [Phenotype: Stable]	Arbes et al. (1999) criteria; % sites with attachment loss ≥ 3 mm (mild PD); gingival and plaque indices assessed. [Chronic PD]	Serum [nephelo- metric method/ Immunohistochemistry]	NM	Hypertension Diabetes mellitus Smoking	Serum Hsp60↑ CRP ↔
	Pejčić (2011) [30]	50	No clinical CAD but focused on CRP as a cardiovascular risk indicator. [Phenotype: Stable]	Moderate–severe chronic PD (≥ 4 sites PD > 5 mm, CAL > 3 mm, ≥ 2 quadrants). [Chronic PD]	Subgingival plaque samples [Radial immunodiffusion]	NM	None – systemic conditions (CVD, DM, etc.) were excluded; participants were systemically healthy	CRP↓ after periodontal therapy
	Saffi (2018) [31]	69	Stable CAD confirmed by ≥ 50% stenosis, prior MI, or revascularization (≥ 6 months). [Phenotype: Stable]	CDC-AAP: ≥2 teeth with PD ≥ 5 mm & CAL ≥ 6 mm Severe PD, ≥ 10 teeth. [Chronic PD]	Blood [Immunoturbidimetry, Multiplex]	NM	Diabetes Smoking Hypertension Dyslipidemia	sVCAM-1↑ sICAM-1↑ P-selectin ↔ CRP ↔
RCT	Lobo (2020) [32]	48	Recent STEMI (confirmed by ECG + biomarkers; 2 weeks post-hospitalization). [Phenotype: Acute]	Severe PD: CAL ≥ 4 mm & PD ≥ 6 mm in ≥ 5 teeth, bleeding in ≥ 8 teeth [Chronic PD]	Blood [ELISA]	NM	Diabetes Smoking Hypertension Dyslipidemia	IL-1β ↔ IL-6 ↔ IL-10 ↔ CRP ↔ Fibrinogen ↔
	Montenegro (2019) [33]	82	Stable CAD diagnosed by Brazilian Society of Cardiology criteria: history of MI, stable angina or ischemia on non-invasive tests, or ≥ 50% lesion in ≥ 1 major coronary artery on angiography. [Phenotype: Stable]	Severe chronic PD (CDC–AAP 2012 criteria): ≥2 teeth with PD ≥ 5 mm and CAL ≥ 6 mm; ≥10 teeth. [Chronic PD]	Blood [Immuno-assay]	97.6% control group were prescribed statin	Diabetes Obesity Hypertension History of MI dyslipidemia; most on statins & aspirin	CRP↓ IL-6↓ IL-8↓ after periodontal therapy
	D’Aiuto et al. (2006) [34]	40	None – participants were systemically healthy (no CAD). [Phenotype: Stable]	≥ 50% dentition with PPD > 4 mm and radiographic alveolar bone loss. (Chronic PD)	Blood [Biochemical Assay]	NM	None – systemic diseases (CVD, DM, infections, etc.) excluded	IL-6 ↓ CRP ↓
	Cullinan et al. (2015) [35]	383	Clinical and medical history data. [Phenotype: Stable]	Periodontal disease severity recorded; 71% had ≥ 1 site PPD ≥ 4 mm (CDC-AAP: 21% PD). [Chronic PD]	Blood [Biochemical Assay]	86% triclosan group were prescribed statins	Diabetes Hypertension Smoking Statin use Anti-inflammatory use	ESR↓ CRP ↔
	Seinost et al. (2020) [36]	90	Peripheral arterial disease (PAD) – all had documented luminal stenosis > 70%. [Phenotype: Stable]	Severe PD (≥ 12 teeth, ≥ 2 sites PPD > 5 mm + CAL > 5 mm + BOP > 20%). [Chronic PD]	Blood/ Gingival crevicular fluid [NM]	76.7% were prescribed statins	Hypertension Diabetes Smoking Statin use	CRP ↔ IL-6 ↔ MMP-8 ↓ oxLDL ↔

↑ significant increase; ↓ significant decrease; ↔ no significant change

*ACS* Acute Coronary Syndrome, *AMI* Acute Myocardial Infarction, *ANG / CA* Angiography / Coronary Angiography, *BMI* Body Mass Index, *BP* Blood Pressure, *BOP* Bleeding on Probing, *CAL* Clinical Attachment Loss, *CAD* Coronary Artery Disease, *CG* Control Group, *CP* Chronic PD, *CRP* C-Reactive Protein, *hs-CRP* High-sensitivity C-reactive protein, *CVD* Cardiovascular Disease, *DM* Diabetes Mellitus, *ESR* Erythrocyte Sedimentation Rate, *ETP* Endogenous Thrombin Potential, *FBS* Fasting Blood Sugar, *FMD* Flow-Mediated Dilation, *GCF* Gingival Crevicular Fluid, *HbA1c* Glycated Hemoglobin, *HDL-C* High-Density Lipoprotein Cholesterol, *HOMA* Homeostatic Model Assessment, *HTN* Hypertension, *ICAM-1 / sICAM-1* (Soluble) Intercellular Adhesion Molecule-1, *IFN-γ* Interferon-gamma, *IL-1β* Interleukin-1 beta, *IL-6* Interleukin-6, *IL-8* Interleukin-8, *IL-10* Interleukin-10, *IPT* Intensive Periodontal Therapy, *LDH* Lactate Dehydrogenase, *LDL-C* Low-Density Lipoprotein Cholesterol, *MMP-8* Matrix Metalloproteinase-8, *MMP-9* Matrix Metalloproteinase-9, *MI* Myocardial Infarction, *MPO* Myeloperoxidase, *NF-κB* Nuclear Factor kappa B, *NM* Not mentioned, *NT-proBNP* N-terminal pro–B-type Natriuretic Peptide, *oxLDL* Oxidized Low-Density Lipoprotein, *PAD* Peripheral Arterial Disease, *PAI-1* Plasminogen Activator Inhibitor-1, *PBMC* Peripheral Blood Mononuclear Cells, *PD* Probing Depth, *PISA* Periodontal Inflamed Surface Area, *PPD* Probing Pocket Depth, *PT* Periodontal Therapy, *PTX3* Pentraxin-3, *RCT* Randomized Controlled Trial, *sVCAM-1 / VCAM-1* (Soluble) Vascular Cell Adhesion Molecule-1, *SRP* Scaling and Root Planing, *STEMI* ST-Elevation Myocardial Infarction, *TC* Total Cholesterol, *TG* Triglycerides, *TIMP-1* Tissue Inhibitor of Metalloproteinase-1, *TNF-α* Tumor Necrosis Factor-alpha

### Meta-analysis

A meta-analysis evaluated the standardised mean differences (SMD) between PD and CAD concerning the associated biomarkers. Studies meeting the predefined inclusion criteria and providing sufficient quantitative data were included in the meta-analysis. The effect sizes (SMD) were calculated for each study, and a random-effects model was used to account for variability between studies. Heterogeneity was assessed using the I² statistic, with values above 50% indicating substantial heterogeneity. The fixed-effect model was also used for comparison, and the pooled effect size was calculated for both models. Subgroup analyses were performed based on the types of studies and biomarkers assessed.

### Risk of Bias (RoB) assessment

The methodological quality of the observational studies included in this review (*n* = 14) was assessed using the Newcastle–Ottawa Scale (NOS) [17], a widely applied tool for evaluating the risk of bias in non-randomised studies. The NOS assesses studies across three domains: (1) Selection of cases and controls, (2) Comparability of study groups, and (3) Exposure assessment. Each item within these domains received a star rating for low risk of bias, with a maximum score of 9 stars. Based on total scores, studies were categorised as follows: low risk of bias (7–9 stars), moderate risk of bias (5–6 stars), and high risk of bias (≤ 4 stars). The results of the NOS assessment are presented in Table 2.

**Table 2:** Newcastle–Ottawa Scale (NOS) for observational studies

Study ID	Representativeness	Non-exposed Selection	Exposure Ascertainment	Outcome Absent at Start	Comparability (Age/Gender)	Comparability (Other)	Outcome Assessment	Follow-up Length	Follow-up Adequacy	Total Score	Overall RoB
Sanikop (2022) [7]	★	★	★	★	★	★	★	★	★	9	Low
Bagavad Gita (2019) [18]	★	★	★	★	★	★	★	★	★	9	Low
Sebring (2024) [19]	★	★	★	★	★	-	★	★	★	8	Low
Górska et al. (2019) [20]	★	★	★	★	★	★	★	-	★	8	Low
Sitompul et al. (2023) [21]	★	★	★	★	★	-	★	★	★	9	Low
Ilango (2023) [22]	★	★	★	★	★	★	★	★	★	9	Low
Teeuw et al. (2015) [23]	★	★	★	★	★	★	-	★	★	8	Low
Joseph et al. (2011) [24]	-	★	★	★	★	★	-	★	★	8	Low
Flores (2014) [25]	-	★	★	★	★	-	★	★	★	7	Low
Fedele (2011) [26]	-	★	★	★	★	★	★	-	★	7	Low
Zanella et al. (2012) [27]	★	★	★	★	★	★	★	-	★	8	Low
Miyoshi et al. (2018) [28]	★	★	★	★	★	★	★	-	★	8	Low
Rizzo et al. (2012) [29]	-	★	★	★	★	★	★	-	★	7	Low
Pejčić (2011) [30]	-	★	★	★	★	★	★	★	★	8	Low

**Fig. 2:**
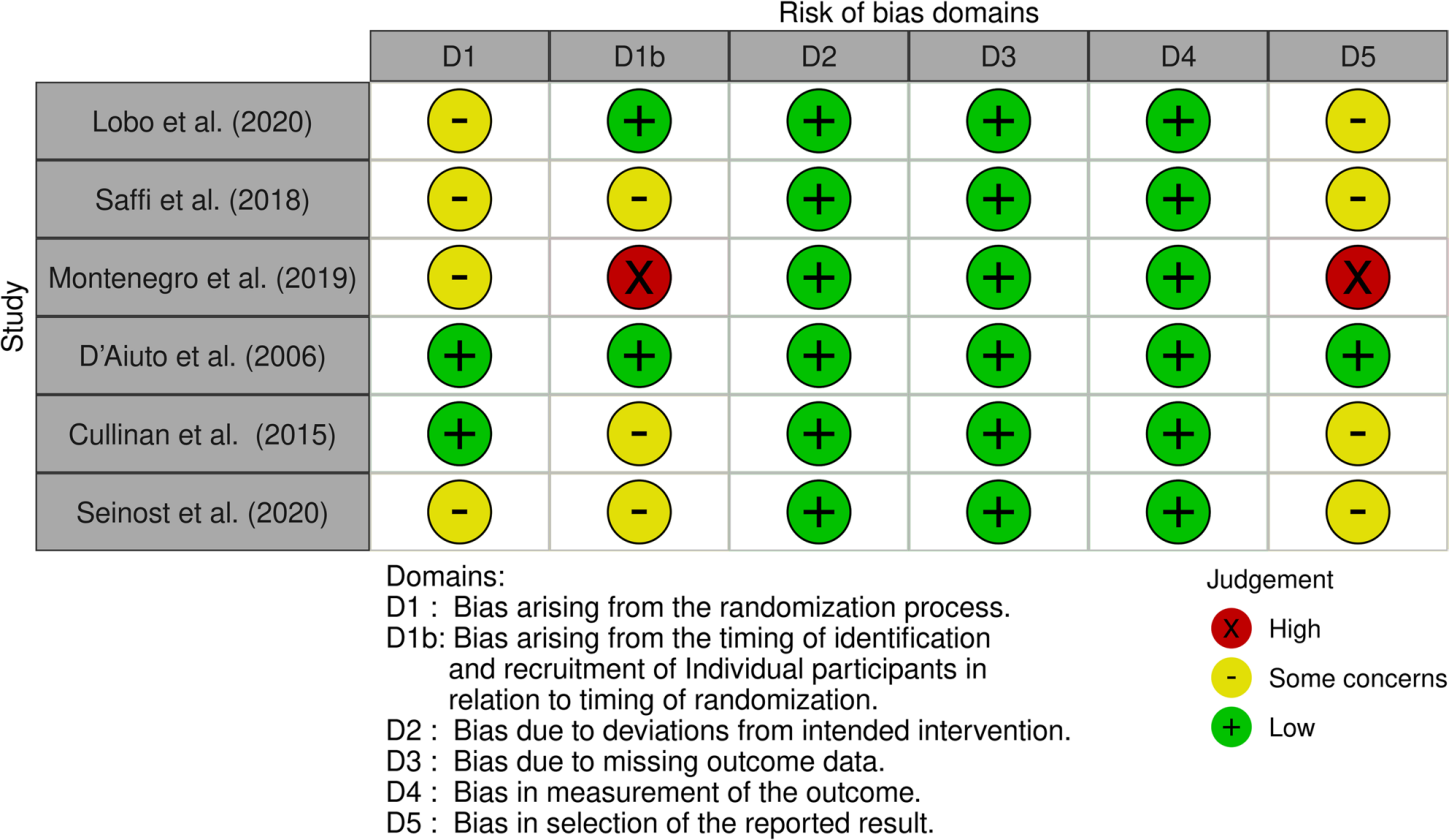
Cochrane RoB 2.0 for RCTs

The methodological quality of the randomised controlled trials (RCTs) included in this review (*n* = 6) was evaluated using the Cochrane Risk of Bias 2.0 (RoB 2.0) tool. RCTs were analysed using the RoB 2.0 platform, as shown in Fig. 2. The RoB 2.0 tool has five domains: (1) bias due to randomisation process; (2) bias due to deviation from planned interventions; (3) bias due to missing outcome data; (4) bias in outcome measurement; and (5) bias in the selection of reported study [37]. Studies were categorised as “low” for low risk of bias, “high” for high risk of bias, “some concern” for uncertain risk, and “no information” for insufficient or missing information.

## Results

### Identification of studies

The systematic search of PubMed, Scopus, and Web of Science yielded a total of 3,245 records. After removing 1,863 records prior to screening, including duplicates, non-English articles, reviews, and studies involving non-human subjects, 1,382 records remained for title and abstract screening. Of these, 1,336 records were excluded based on title and abstract assessment, leaving 46 reports for retrieval. Five reports were not retrieved, resulting in 41 full-text articles assessed for eligibility.

Following full-text evaluation, 21 studies were excluded due to reasons including non-relevant populations, absence of confirmed CAD diagnosis, or lack of focus on the periodontitis–CAD relationship. Ultimately, 20 studies met the inclusion criteria and were included in the qualitative synthesis. The study selection process is illustrated in the PRISMA flow diagram (Fig. 1).

### Overview of biomarkers in PD and CAD or cardiovascular risk–related studies

This systematic review and meta-analysis included 20 studies. Table 1 provides a comprehensive summary of the included studies, study design, sample characteristics, diagnostic criteria, biological samples, assay methods, and the reported associations between PD and systemic inflammatory biomarkers in individuals with CAD or related cardiovascular risk indicators. Most studies compared individuals with cardiovascular conditions or risk indicators and PD to healthy controls regarding biomarker and disease associations. These studies often assess inflammatory markers such as CRP and IL-6, followed by IL-1β, IL-8, and TNF-α. The observational studies provide a snapshot of the biomarker values and characteristics of PD present at that time. Additionally, RCTs were analysed, which is the highest level in clinical research. These trials examine the effect of targeted interventions on biomarkers in patients with CVD or related inflammatory risk and PD, and are therefore able to provide more substantial evidence of causation.

The studies were categorised into two distinct clinical groups based on the cardiovascular state. Four studies evaluated patients during an acute cardiac event, while the remaining 16 studies reported on patients with stable CAD. On the other hand, in the PD state, 20 studies focused on subjects diagnosed with chronic periodontitis. Although these are clinically distinct conditions, both represent chronic oral inflammatory infections that may contribute to systemic inflammatory responses associated with CAD.

Regardless of the study design, our results reveal that PD has a statistically significant but varied association with systemic inflammatory biomarker profiles, particularly increased levels of CRP and IL-6, as well as lipid perturbations. Collectively, these studies further support the association between PD and CVD, highlighting the importance of biomarkers in understanding the interrelationship between these two conditions.

### Key biomarkers in the PD-CAD connection

The twenty studies included in this review examined different biomarkers; some studies focused on a single biomarker, while some focused on multiple biomarkers. Table 3 presents the five most frequently reported biomarkers associated with PD and CAD. CRP emerged as the most widely reported biomarker, appearing in 14 studies, which highlights its potential key role in inflammation and its potential as a key biomarker related to PD and CAD. High-sensitivity CRP (hs-CRP), a more sensitive assay capable of detecting low-grade inflammation at levels ranging from 0.5 to 10 mg/L (compared to 10–1000 mg/L for conventional CRP), was also reported in 3 studies. As both represent the same protein, CRP and hs-CRP were grouped in the present review to provide a more precise representation of overall CRP involvement while acknowledging their difference in analytical sensitivity. Following closely are IL-6, which was mentioned in 8 studies, indicating the vital role of lipid metabolism and inflammatory processes in both diseases. The remaining biomarkers, including IL-1β, IL-8, and TNF-α, were less frequently investigated, which may indicate more limited and variable evidence, but still play a potential role in the association between PD and CAD. These data underscore the key biomarkers that have been central to the concept of shared mechanisms of inflammation and lipid metabolism between the two diseases.

**Table 3:** Top 5 biomarkers

Biomarkers	Frequency
CRP (CRP, hs CRP)	14 (14,3)
IL-6	8
IL-1β	3
IL-8	2
TNF-α	1

It is crucial to differentiate between the prevalence of a biomarker in literature and its statistical significance as a contributing factor. The descriptive synthesis shown in Table 3 reveals that CRP and IL-6 were the most commonly reported as indicators of research trends compared to that measure of clinical effect size. To validate the robustness of the conclusions, the meta-analytical findings were prioritized which unbiased evaluate studies based on their sample size and variance.

### Quantitative meta-analysis

From the selected 20 studies, a subset was eligible for quantitative meta-analysis. Studies that were included in the meta-analysis include (i) the most frequent biomarkers discussed, (ii) provide sufficient statistical information to compute SMD, including mean, standard deviations and samples sizes besides and (iii) implemented comparable outcome definitions and measurement units to allow pooling across studies. As for exclusion from meta-analysis, studies were ruled out if they only report qualitative findings and did not provide sufficient numerical data to calculate effect size. Based on these criteria, five studies were included in the analysis of CRP levels, while three studies were included in the analysis of IL-6 levels.

### CRP levels in PD-CAD individuals

**Fig. 3:**
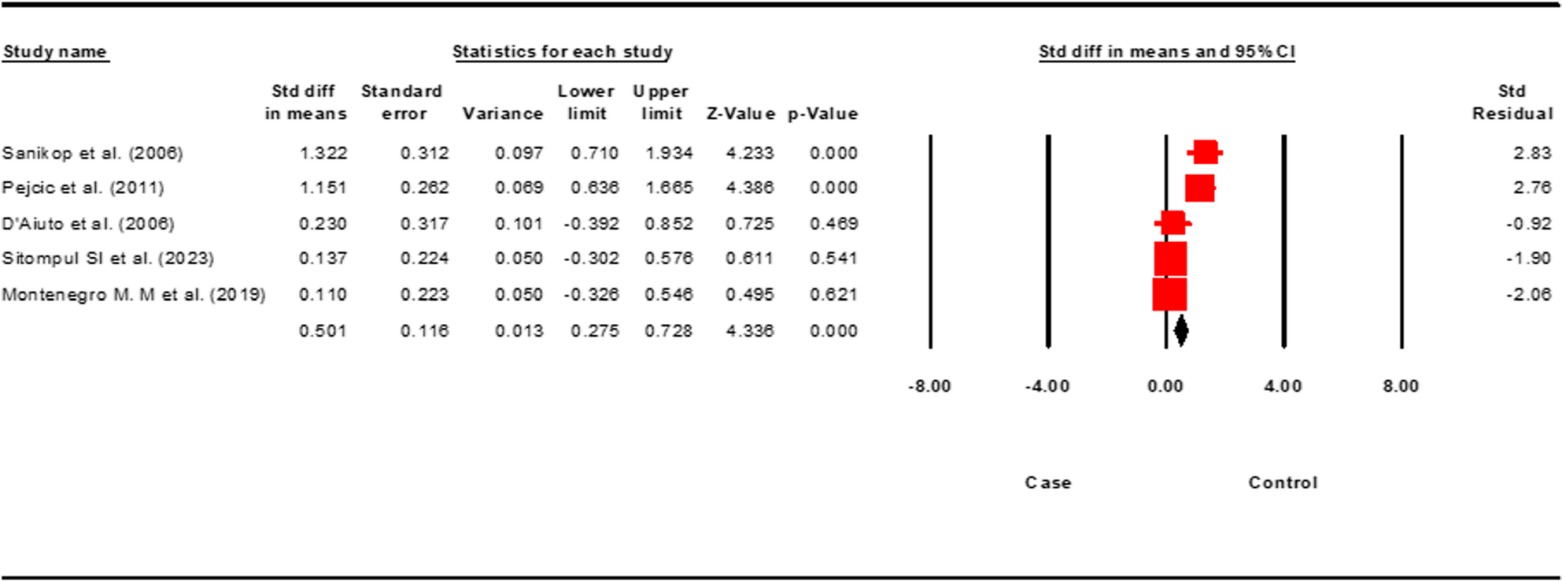
Meta-analysis of CRP levels in PD-CAD individuals based on intergroup comparison. Forest plot showing standardized mean differences (SMD) with 95% confidence intervals. Test for overall effect: SMD = 0.501, *p* < 0.001; Test for heterogeneity: Q = 19.50, df = 4, *p* = 0.001

The updated meta-analysis incorporated five studies to estimate the pooled SMD in relation to baseline CRP level among different patient groups (Fig. 3). Under the fixed-effects model, the pooled effect size was 0.501 (SE = 0.116; 95% CI: 0.275–0.728), yielding a statistically significant overall effect (Z = 4.336, *p* < 0.001). However, given the presence of substantial heterogeneity (Q = 19.504, df = 4, *p* = 0.001; I² = 79.49%), the random-effects model was also applied. Under this model, the pooled effect size increased to 0.573 (SE = 0.259; 95% CI: 0.065–1.082), remaining statistically significant (Z = 2.209, *p* = 0.027). The heterogeneity estimate was substantial, with a tau-squared value of 0.265 (tau = 0.515), further reinforcing the appropriateness of the random-effects approach due to between-study variance. The corresponding forest plot visually supported these findings, illustrating the variability in effect sizes across studies and the broader confidence interval around the random effects estimate. Notably, while the fixed-effects model suggests a moderate and robust overall effect, the random-effects model, which accounts for inter-study heterogeneity, presents a more conservative estimate with reduced precision. Therefore, the findings should be interpreted within the context of substantial heterogeneity, and the random-effects model provides a more appropriate summary estimate in this case.

**Fig. 4:**
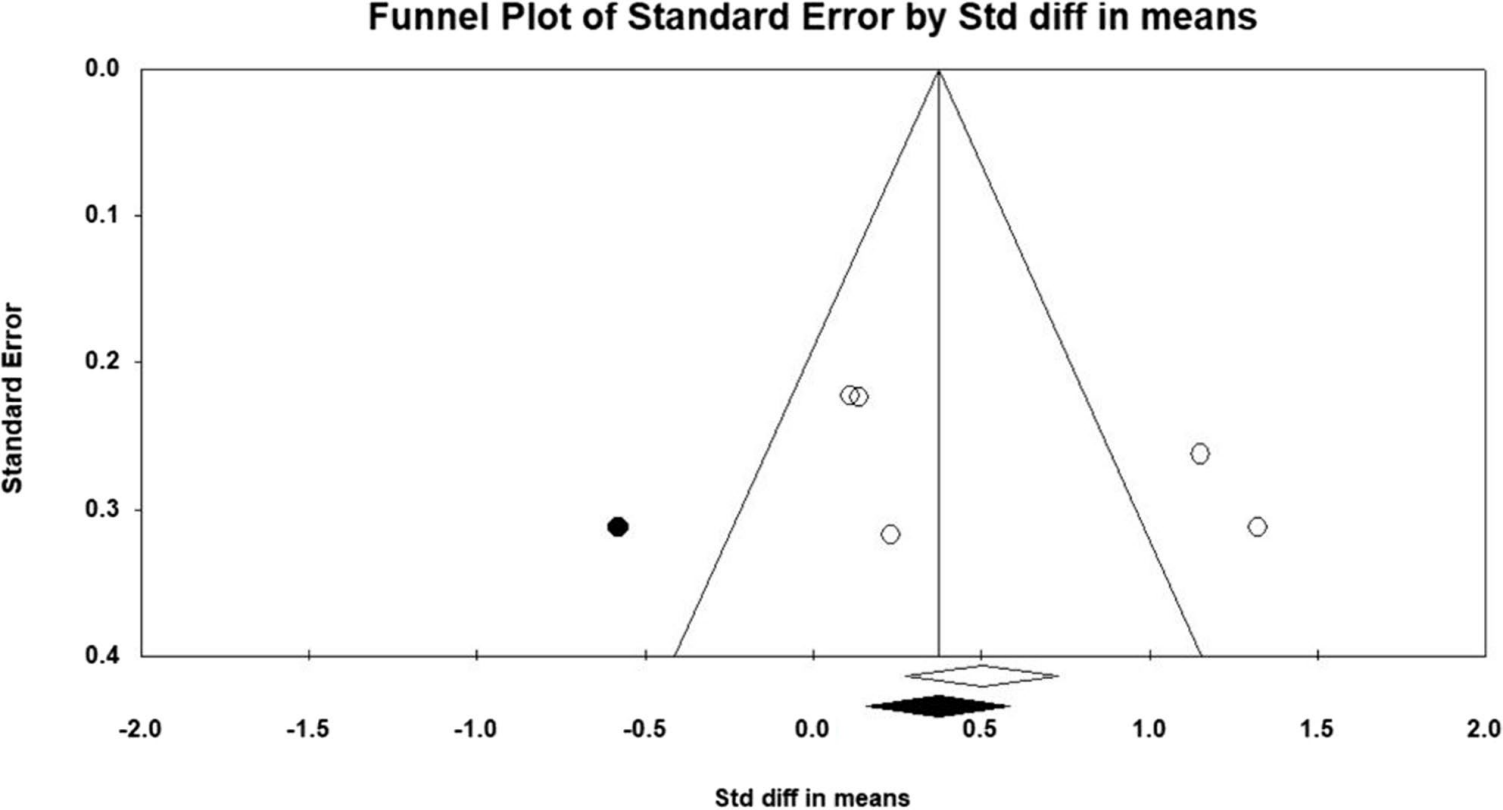
Funnel plot for assessing publication bias in the level of CRP in PD-CAD individuals. ● = plot imputed, ○ = plot observed. Egger’s linear regression test (Intercept = 7.630, t = 1.239, *p* = 0.303)

Visual inspection of the funnel plot revealed minor asymmetry, with one study positioned outside the pseudo 95% confidence region on the left side of the mean, suggesting the potential presence of small-study effects or missing studies with negative or null findings (Fig. 4). Duval and Tweedie’s trim-and-fill procedure identified one potentially missing study to the left of the mean, indicating slight asymmetry. After imputing this study, the adjusted overall effect size decreased from 0.5011 to 0.3708 under the fixed-effects model, and from 0.5731 to 0.3930 under the random-effects model. While both adjusted estimates remained positive, the reduction suggests that the original pooled effect may be somewhat overestimated due to potential publication bias. Egger’s regression intercept test produced a non-significant intercept of 7.630 (SE = 6.156, t = 1.239, *p* = 0.303 two-tailed), further supporting the absence of statistically significant small-study effects. Despite a relatively high intercept point estimate, the wide confidence interval (− 11.96 to 27.22) reflects imprecision and limited power due to the small number of studies. Taken together, the results of statistical tests, visual inspection, and imputation-based sensitivity analysis suggest that, while there is some indication of minor publication bias, particularly through the trim-and-fill adjustment, the overall evidence does not support the presence of substantial or systematic bias. Nevertheless, the modest number of included studies limits the statistical power of these tests, and the possibility of small-study effects cannot be entirely excluded. These considerations should be taken into account when interpreting the pooled effect estimates.

### IL-6 levels in PD-CAD individuals

**Fig. 5:**
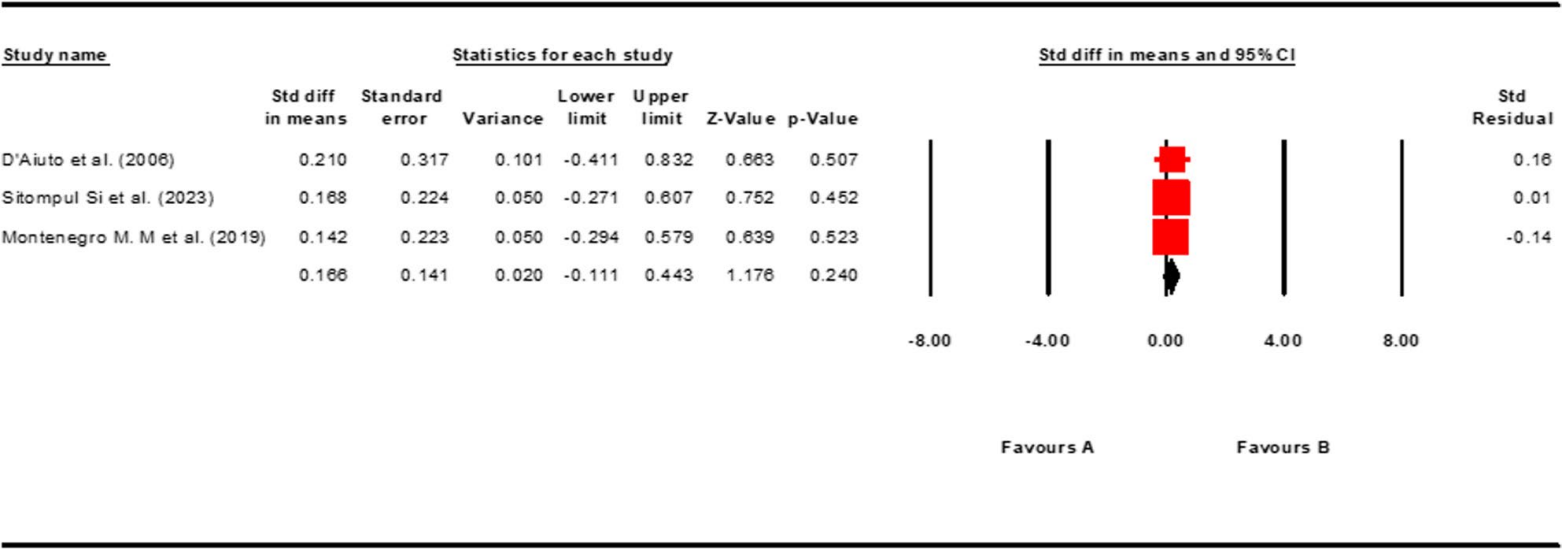
Meta-analysis of IL-6 levels in PD-CAD individuals based on intergroup comparison. Forest plot showing standardized mean differences (SMD) with 95% confidence intervals. Test for overall effect: SMD = 0.166, *p* = 0.240; Test for heterogeneity: Q = 0.031, df = 2, *p* = 0.985

The meta-analysis synthesised data from three independent studies examining the SMD between case and control groups (Fig. 5). Under both fixed-effects and random-effects models, the pooled effect size was 0.166, with a standard error of 0.141 and a variance of 0.020. The 95% CI ranged from − 0.111 to 0.443, indicating that the overall effect was not statistically significant (Z = 1.176, *p* = 0.240). This finding was consistent across both analytical models, reflecting identical numerical outputs due to the absence of between-study heterogeneity. Heterogeneity analysis yielded a Q-value of 0.031 (df = 2, *p* = 0.985), with an I² statistic of 0.0%, confirming negligible heterogeneity across the included studies. The Tau-squared was 0.000, further supporting the homogeneity of effect sizes. The forest plot visually reflected the consistency in effect sizes, with all confidence intervals overlapping the null line. These results indicate that there is no statistically significant overall benefit of the case interventions over the control conditions in the analyzed studies, and the minimal heterogeneity justifies the robustness of the pooled estimate.

**Fig. 6:**
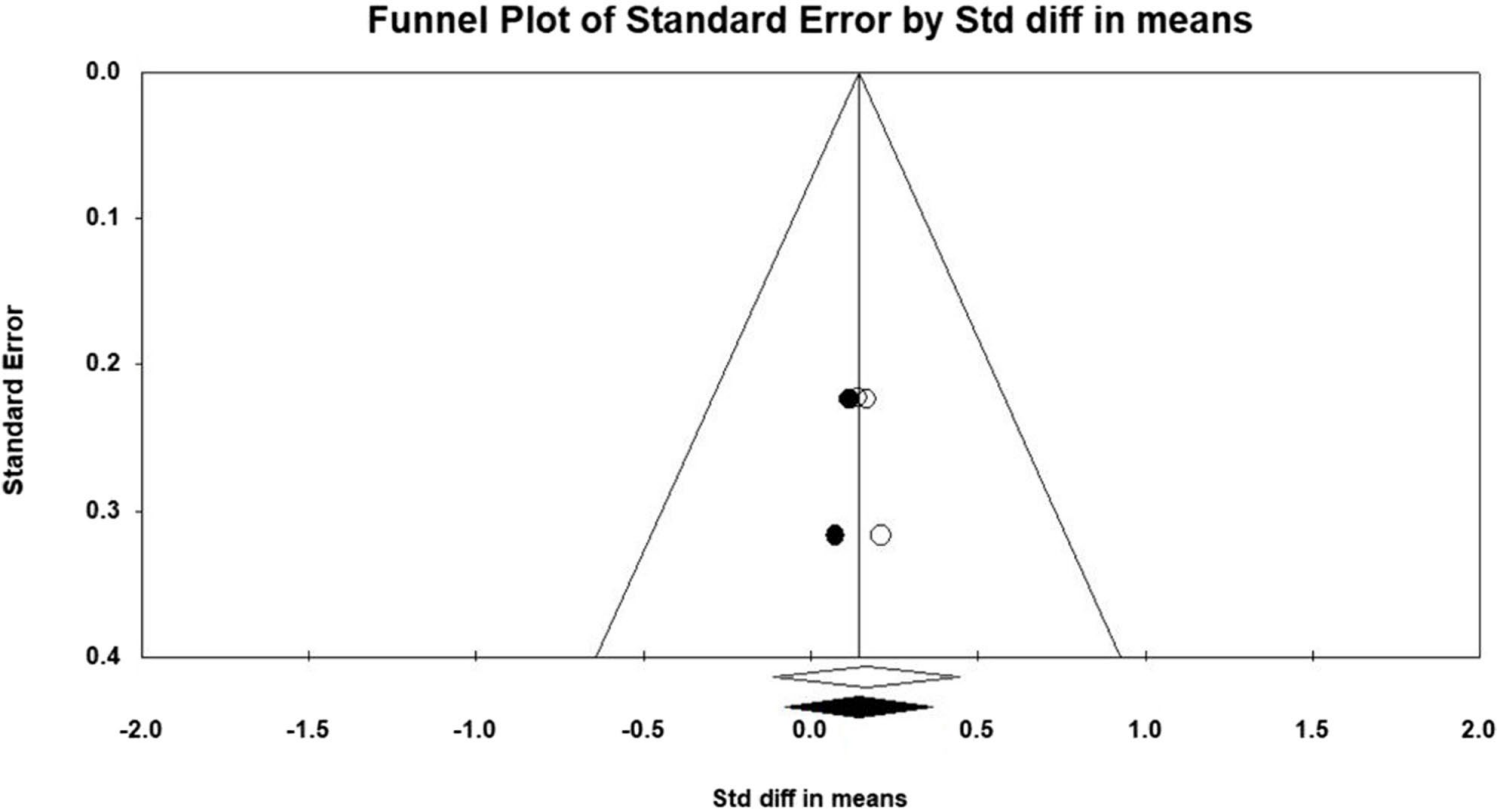
Funnel plot for assessing publication bias in the IL-6 levels in PD-CAD individuals. ● = plot imputed, ○ = plot observed. Egger’s linear regression test (Intercept = 0.591, t = 0.302, *p* = 0.300)

Egger’s regression intercept analysis yielded an intercept of 0.5908 (SE = 0.3015) with a two-tailed *p*-value of 0.3004, suggesting no statistically significant small-study effects. The funnel plot, which graphically displays the standard error against the SMD, appeared symmetrical, further supporting the absence of substantial publication bias (Fig. 6). To explore the potential impact of unpublished studies, Duval and Tweedie’s trim-and-fill analysis was applied. The method estimated two potentially missing studies to the left of the mean. After adjustment, the pooled effect size under both fixed- and random-effects models slightly decreased from 0.1662 to 0.1424, with the 95% CI narrowing slightly from − 0.1109 to 0.4433 (unadjusted) to − 0.0769 to 0.3616 (adjusted), indicating a minimal shift in the overall effect estimate. Taken together, these findings suggest minimal risk of publication bias in the present meta-analysis. Although the number of included studies was limited, the convergence of statistical tests, visual assessments, and sensitivity analyses provides consistent evidence that the synthesised results were not meaningfully influenced by selective reporting or small-study effects.

### Risk of Bias (RoB)

The overall risk of bias among the 20 included studies varied due to differences in study design and reporting quality. Observational studies (*n* = 14; NOS) demonstrated high methodological quality, with seven studies rated as low risk of bias (7–9 stars). Minor issues were identified in the comparability section, where some studies were unable to account for all major confounders, and in the exposure/outcome evaluation, where CAD confirmation methodologies were not always fully described.

In addition, randomised controlled trials (*n* = 6, RoB = 2.0) indicated that most randomised controlled trials presented some concerns, primarily due to unclear randomization procedures and potential deviations from intended interventions, particularly related to concurrent statin therapy. One study was rated as a high risk of bias due to a significant imbalance in co-interventions, while only one study demonstrated a low overall risk of bias.

## Discussion

This systematic review presents a comprehensive evaluation of the association between PD and biomarker profiles in adults diagnosed with CAD. Through an extensive literature search and critical appraisal of 20 clinical studies, several studies reported associations between periodontal inflammation and systemic biomarkers associated with CAD, although the strength and significance of these findings varied across studies. The most frequently studied biomarker was CRP, reported in nearly half of the studies, followed by IL-6, TNF-α, and other inflammatory mediators, including IL-1β, IL-8, and hs-CRP. These biomarkers were often elevated in patients suffering from both PD and CAD, which may reflect a shared inflammatory profile linking oral and cardiovascular conditions.

The findings of this systematic review add to the growing body of evidence suggesting a potential biological link between PD and CAD, which may be partly explained by systemic inflammation. Among the reviewed studies, several reported elevated levels of inflammatory biomarkers, including CRP, IL-6, TNF-α, and IL-1β, in patients with concurrent periodontal disease and cardiovascular conditions. CRP was the most frequently examined marker, with multiple studies reporting higher level in individuals affected by both conditions [6, 20, 23, 27]. Similarly, IL-6 and TNF-α were reported to be upregulated in several cohorts, suggesting a possible role for these cytokines in the shared pathophysiological mechanisms [26, 34, 36]. However, variability across studies and the presence of heterogeneity indicate that these associations should be interpreted with caution.

Incorporating the findings from the meta-analysis, five studies were included in the primary pooled meta-analysis for CRP analyses that also showed a significant pooled effect (SMD = 0.501; *p* = 0.001), while three studies stated that IL-6 showed no significant association (SMD = 0.166; *p* = 0.240). These findings are consistent with previous observational research reporting association between PD and CVD, suggesting that periodontal inflammation may exacerbate systemic inflammation, leading to increased cardiovascular risk [38, 39]. The meta-analysis suggests that systemic biomarkers, particularly CRP, IL-6, and TNF-α, although the magnitude and statistical significance of these associations varied across studies [40, 41]. These inflammatory markers have been proposed as potential mediators in the relationship between these two conditions, reflecting the systemic inflammatory burden associated with PD [42].

Substantial heterogeneity was observed in the levels of CRP in PD-CAD individuals (I²=79.49%). This variability arises from differences in diagnostic criteria for both PD (e.g., CAL > 4 mm, PPD > 5 mm at varying site thresholds, or radiographic alveolar bone loss) and CAD (e.g., > 50–70% stenosis, involvement of ≥ 1 coronary artery, or history of MI). In addition, methodological differences in biomarker assessment contribute to heterogeneity, including variation in assay types (e.g., ELISA, radial immunodiffusion, immunoturbidimetry) and sample sources (serum, plasma, saliva, subgingival plaque, and atheromatous plaque). Population-level factors further contribute to heterogeneity, including consistent reporting of statin and aspirin use (10 studies not reported; 6 reporting statin use; 2 aspirin use), as well as differences in comorbidity profiles. While 5 studies excluded any comorbidities, 11 included participants with risk factors such as smoking and diabetes, which may influence inflammatory biomarker levels. These methodological and clinical variations, which were summarized in Table 1, likely underpin the observed heterogeneity and should be considered when interpreting the pooled estimates.

Interestingly, a study by Joseph et al. (2011) reported relatively larger effect sizes, suggesting a possible association between PD and CAD, particularly when considering inflammatory markers like CRP [23]. These findings may support the hypothesis that chronic periodontal infection could contribute to systemic inflammation, which may be associated with an increased risk of cardiovascular events [43]. The meta-analysis further corroborates the notion that systemic inflammation is a critical mechanism linking PD to CAD, underscoring the clinical relevance of monitoring inflammatory biomarkers such as CRP, IL-6, and TNF-α in patients with periodontal disease [44].

These findings support the hypothesis that systemic inflammation, rather than isolated gene or protein expression, serves as a functional link between periodontal and cardiovascular pathology. For example, IL-6 was not only elevated in patients with coexisting CAD and PD [19], but also shown to correlate with CRP levels, suggesting a downstream cascade with systemic consequences [18]. Furthermore, studies examining molecular targets such as VCAM-1, P-selectin, and soluble Receptor for Advanced Glycation End Products (sRAGE) suggest involvement of endothelial dysfunction and immune activation in mediating this association [31, 33].

Interestingly, multiple RCTs have reported reductions in systemic inflammatory markers following periodontal therapy [30, 31, 34], suggesting that systemic inflammatory profiles associated with PD may be modifiable. These therapeutic responses further affirm the clinical relevance of targeting periodontal inflammation as a strategy to mitigate cardiovascular burden. Additionally, the identification of molecular signatures such as Pentraxin-3 and MMP-8 in CAD patients with PD suggests the existence of shared genetic or proteomic regulators [19, 36]. Altogether, the synthesis of biomarker trends across studies indicates possible similarities in inflammatory patterns, which may suggest potential overlap in therapeutic and diagnostic strategies between periodontology and cardiology.

The results of this review suggest that systemic inflammatory biomarkers, particularly CRP, IL-6, and TNF-α, may provide clinical insight into the interconnection between PD and CAD [6, 26, 34]. The reported elevation of these markers highlights the role of chronic oral inflammation in systemic vascular dysfunction. Moreover, some studies have reported reductions in these biomarkers following periodontal therapy underscore the potential for periodontal intervention as an adjunct to cardiovascular risk management [30, 31]. These findings support a multidisciplinary approach that integrates oral health into CAD prevention strategies, particularly for high-risk patients. The convergence of inflammatory pathways further justifies the use of biomarker-based screening tools and strengthens the rationale for collaborative care models that link periodontology and cardiology.

Nevertheless, the findings from Egger’s regression intercept analysis, Duval and Tweedie’s trim-and-fill method, and funnel plot should be interpreted with caution because statistical methods for detecting publication bias may have limited reliability when the number of included studies is small. Therefore, although the analyses did not indicate substantial publication bias, the possibility of undetected bias cannot be entirely excluded. This limitation should be considered when interpreting the pooled results of the present meta-analysis.

### Limitation

Several limitations should be acknowledged. Most of the included studies were observational, which limits the ability to infer causal relationships between PD and CAD. Considerable heterogeneity was also observed across studies in terms of periodontal diagnostic criteria, biomarker assessment methods, and study populations. In addition, only a small number of studies were available for certain meta-analyses, particularly for IL-6, which may have limited statistical power. Potential confounding factors, such as smoking status, medication use, and comorbidities, were not consistently controlled across studies. Therefore, the findings should be interpreted with caution.

### Future directions

Future studies should prioritise well-designed longitudinal and interventional research to clarify the temporal relationship between periodontal inflammation and cardiovascular biomarkers. Standardisation of periodontal disease definitions, biomarker assay methods, and control of confounding factors such as lifestyle and pharmacological influences are essential to improve comparability across studies. In addition, larger multicentre studies and clinical trials evaluating the impact of periodontal therapy on systemic inflammation and cardiovascular outcomes would further strengthen the evidence base. The integration of omics technologies and machine learning approaches may further enhance biomarker discovery and improve risk stratification across diverse populations.

## Conclusion

In conclusion, this systematic review summarizes current evidence examining the relationship between chronic PD and systemic inflammatory biomarkers among individuals with CAD. Overall, several studies reported elevated levels of inflammatory biomarkers, particularly CRP, IL-6, and TNF-α, in patients with coexisting chronic PD and CAD. However, the magnitude and statistical significance of these associations varied across studies; heterogeneity in study design, populations, and biomarker assessments warrants cautious interpretation. The findings suggest that periodontal inflammation may contribute to systemic inflammatory responses relevant to cardiovascular pathology, although the current evidence primarily supports an association rather than a causal relationship. While some studies reported reductions in inflammatory markers following periodontal therapy, further well-designed longitudinal and interventional studies are required to clarify the directionality and clinical significance of this relationship.

## Supplementary Information

Supplementary Material 1.

## Data Availability

All data generated and analysed during this study are included in this article or are available from the corresponding author upon reasonable request.

## Abbreviations

ACS: Acute Coronary Syndrome
AMI: Acute Myocardial Infarction
BOP: Bleeding on Probing
CAD: Coronary Artery Disease
CAL: Clinical Attachment Loss
CRP: C-Reactive Protein
CVD: Cardiovascular Disease
IL-1β: Interleukin-1 beta
IL-6: Interleukin-6
IL-8: Interleukin-8
NOS: Newcastle–Ottawa Scale
PD: Periodontitis
PPD: Probing Pocket Depth
PRISMA: Preferred Reporting Items for Systematic Reviews and Meta-Analyses
RCT: Randomized Controlled Trial
SMD: Standardised Mean Difference
TNF-α: Tumor Necrosis Factor-alpha
